# Liver Transcriptome Analysis of the Large Yellow Croaker (*Larimichthys crocea*) during Fasting by Using RNA-Seq

**DOI:** 10.1371/journal.pone.0150240

**Published:** 2016-03-11

**Authors:** Baoying Qian, Liangyi Xue, Hongli Huang

**Affiliations:** 1 College of Marine Sciences, Ningbo University, Ningbo, Zhejiang, China; 2 School of life science, Taizhou University, Taizhou, Zhejiang, China; 3 Collaborative Innovation Center for Zhejiang Marine High-Efficiency and Healthy Aquaculture, Ningbo University, Ningbo, Zhejiang, China; International Centre for Genetic Engineering and Biotechnology, ITALY

## Abstract

The large yellow croaker (*Larimichthys crocea*) is an economically important fish species in Chinese mariculture industry. To understand the molecular basis underlying the response to fasting, Illumina HiSeq^TM^ 2000 was used to analyze the liver transcriptome of fasting large yellow croakers. A total of 54,933,550 clean reads were obtained and assembled into 110,364 contigs. Annotation to the NCBI database identified a total of 38,728 unigenes, of which 19,654 were classified into Gene Ontology and 22,683 were found in Kyoto Encyclopedia of Genes and Genomes (KEGG). Comparative analysis of the expression profiles between fasting fish and normal-feeding fish identified a total of 7,623 differentially expressed genes (*P* < 0.05), including 2,500 upregulated genes and 5,123 downregulated genes. Dramatic differences were observed in the genes involved in metabolic pathways such as fat digestion and absorption, citrate cycle, and glycolysis/gluconeogenesis, and the similar results were also found in the transcriptome of skeletal muscle. Further qPCR analysis confirmed that the genes encoding the factors involved in those pathways significantly changed in terms of expression levels. The results of the present study provide insights into the molecular mechanisms underlying the metabolic response of the large yellow croaker to fasting as well as identified areas that require further investigation.

## Introduction

Several single net-cage-farmed marine fish, including the large yellow croaker (*Larimichthys crocea*), undergo alternating periods of fasting and feeding during their life cycle, which is mainly due to the temperature changes in the aquatic environment [1]. In the winter, this fish species would undergo fasting for almost three months. The fasting condition is strongly associated with changes in lipid metabolism and storage, as well as mobilization of body energy reserves [2], particularly those involving the liver, which is an indicator organ of the nutritional and physiological status of an organism [3]. The liver is also the first organ that is affected when fish are subjected to food deprivation [4]. Several changes occur during fasting and re-feeding of teleosts [5–7], which include enzymatic activities, hormone levels, and protein synthesis or protein turnover [8–11].

In recent years, the development of the transcriptome sequencing technology and progress in bioinformatics, particularly in the development of *de novo* assembly tools, have provided an unprecedented increase in transcriptome data on gene expression, biological pathways, and molecular mechanisms [12–17]. Transcriptome assembly and annotation has been conducted in various fish species such as the rainbow trout (*Salmo gairdneri*) [18], zebrafish (*Danio rerio*) [19], sea bass (*Lateolabrax japonicas*) [20], steelhead (*Oncorhynchus mykiss*) [21], and sea bream (*Sparus aurata*) [22]. Using a large yellow croaker spleen, Mu *et al*. detected changes in immune system-relevant genes, as well as in several signaling pathways involved in immunity during infection of *Aeromonas hydrophila* [23] and the induction of polyriboinosinic:polyribocytidylic acid [24]. Xiao *et al*. studied the comprehensive transcriptome from multi-tissue mixture of the large yellow croaker [25]. Studies on the transcriptome of the large yellow croaker have been previously conducted, but these mainly focused on immunity. There are currently no reports on the use of *de novo* RNA-seq technology to analyze changes in the transcriptome profile of this fish during fasting.

The large yellow croaker is an economically important marine fish in China, and its annual output is the largest among any other single net-cage-farmed marine species [23, 24]. This fish species naturally undergoes fasting in the summer and winter of each year as a response to the respective high or low temperature of seawater. Therefore, this fish utilizes liver fat and hepatic glycogen to generate energy during fasting seasons, and consequently stores liver glycogen and synthesizes liver fat when re-feeding. In the present study, the large yellow croaker was used as a model to investigate the molecular response underlying the fasting condition. To further understand the molecular basis of this particular metabolic reaction and to explore the key genes and pathways of fat metabolism and the glycometabolism in the large yellow croaker, the Illumina HiSeq^TM^ 2000 sequencing technology was used to conduct a transcriptome profiling analysis of this fish. A transcriptome database containing 38,728 identified unigenes were obtained, and the unigenes that were found to be involved in the fat metabolism, glycometabolism, and protein metabolism pathways were further analyzed. A comparative gene transcription analysis of livers from fasting fish and normal-feeding fish was performed using the RNA-seq technology to investigate its differential transcriptomic profile. Furthermore, real-time PCR was used to analyze the changes in expression of metabolically relevant genes encoding for key enzymes involved in fasting and re-feeding.

## Results

### RNA-seq of liver transcriptome

**Fig 1:**
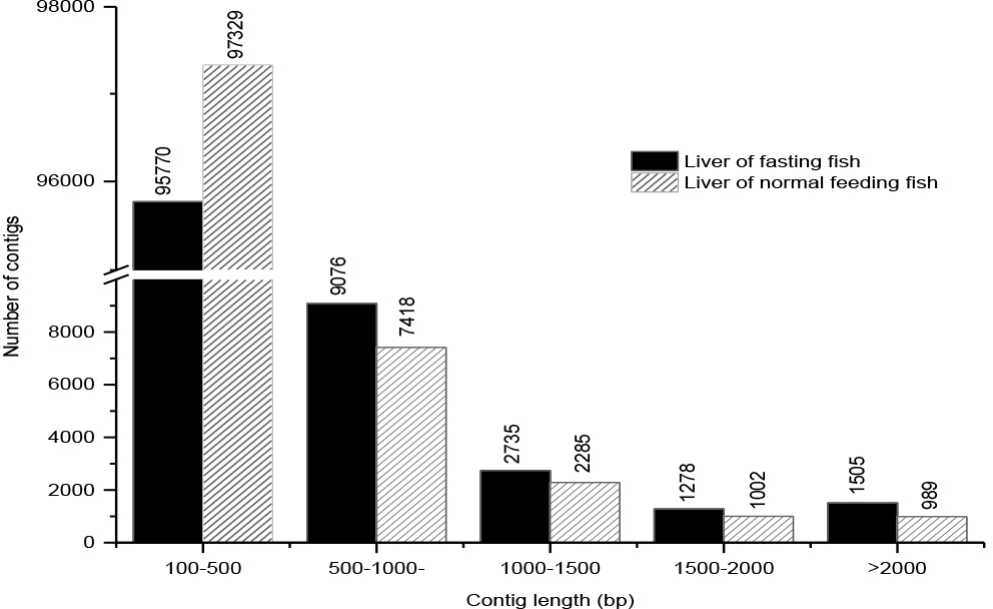
Length statistics of contigs obtained from the two livers of large yellow croaker transcriptome libraries. The length distribution of the transcriptome libraries is shown. Sequences with lengths of 100–500 bp were the most abundant in the two transcriptome libraries, encompassing 86.78% and 89.27% of the contigs in the transcriptome library of fasting fish and normal feeding fish, respectively.

To better understand the molecular changes in the metabolic response of the fasting large yellow croaker, two Solexa cDNA libraries were constructed from the livers of fasting and normal feeding large yellow croaker. After conducting replicate experiments and ensuring quality control, Illumina HiSeq^TM^ 2000 was used in sequencing the cDNA libraries. High-throughput paired-end sequencing yielded 60,090,728 and 57,121,620 total raw reads from the livers of fasting fish and normal feeding fish, respectively. Then, 54,933,550 and 52,848,276 clean reads were obtained after filtering the raw reads. The clean reads were assembled into 110,364 and 109,023 contigs, respectively, using the short oligonucleotide analysis package (SOAP) *de novo* software (**Fig 1**), respectively. Ultimately, 60,998 unigenes were annotated from 110,364 contigs, and 59,671 unigenes were annotated from 109,023 contigs.

### Analysis of liver differential expression profiles between fasting and normal-feeding large yellow croakers

To identify the differentially expressed genes, the liver transcriptome data of the large yellow croakers at 21 days after fasting and normal feeding were analyzed by using fragments per kb per million fragments (FPKM) [26]. The criteria of a two-fold or greater change in expression and *P* < 0.05 were selected to identify the significantly upregulated or downregulated genes during fasting. Using this criteria, a total of 7,623 differentially expressed genes were detected, including 2,500 upregulated genes and 5,123 downregulated genes (S1 Table).

To generate an overview of the functions of the differentially expressed genes identified by FPKM, Gene Ontology (GO) analysis of these genes was performed using GO-TermFinder. According to the GO terms, 8,487 genes were classified into three major functional categories, including 2937 genes in biological process, 2699 genes in cellular component, and 2851 genes in molecular function. The terms from the function ontology with a *P* ≥ 1 in each category were distributed across more than 100 subcategories. The differentially expressed genes in the biological processes were determined to be mainly related to protein metabolism, fat metabolism, glycometabolism, and nucleic acid metabolism. The main subcategory of the cellular component was the cytoplasmic part, and in the category of molecular function, the most significant subcategory was oxidoreductase activity.

To identify the biological pathways that were active in the large yellow croaker at 21-d fasting fish, 7,623 differentially expressed genes were mapped to canonical signaling pathways that were found in the Kyoto Encyclopedia of Genes and Genomes (KEGG). Ultimately, a total of 3,859 genes were mapped to 67 statistically significant categories (*P* < 0.05). The top 33 pathways are summarized in Table 1. Among these pathways, protein processing in the endoplasmic reticulum was represented by 44 downregulated genes. Six upregulated genes and 7 downregulated genes were involved in pyruvate metabolism. Several genes were also determined to be involved in major metabolic-related pathways such as fat digestion and absorption pathway, protein export pathway, citrate cycle (TCA cycle), and glycolysis/gluconeogenesis pathway.

**Table 1:** Statistically significant KEGG classifications of large yellow croaker genes.

Pathway	Gene No.(%*)	P value	Pathway ID
Metabolic pathways	716 (17.68%)	9.00367e-31	ko01100
Complement and coagulation cascades	119 (2.94%)	5.214812e-13	ko04610
Protein processing in endoplasmic reticulum	157 (3.88%)	1.618794e-12	ko04141
Ribosome	87 (2.15%)	3.504371e-12	ko03010
Pyruvate metabolism	49 (1.21%)	1.561337e-09	ko00620
Arachidonic acid metabolism	49 (1.21%)	2.196846e-09	ko00590
Linoleic acid metabolism	40 (0.99%)	4.655279e-09	ko00591
Fat digestion and absorption	51 (1.26%)	5.5317e-09	ko04975
Proteasome	32 (0.79%)	1.89458e-08	ko03050
Protein export	20 (0.49%)	2.545384e-08	ko03060
Terpenoid backbone biosynthesis	22 (0.54%)	7.123303e-08	ko00900
Propanoate metabolism	39 (0.96%)	5.035928e-07	ko00640
Steroid biosynthesis	23 (0.57%)	1.735558e-06	ko00100
Vitamin digestion and absorption	32 (0.79%)	5.084006e-06	ko04977
Synthesis and degradation of ketone bodies	12 (0.3%)	6.549285e-06	ko00072
Pentose and glucuronate interconversions	30 (0.74%)	1.030834e-05	ko00040
Glycine, serine and threonine metabolism	44 (1.09%)	1.316588e-05	ko00260
Steroid hormone biosynthesis	30 (0.74%)	1.390942e-05	ko00140
Tryptophan metabolism	38 (0.94%)	1.587954e-05	ko00380
Fatty acid metabolism	40 (0.99%)	1.633000e-05	ko00071
Valine, leucine and isoleucine degradation	39 (0.96%)	2.03564e-05	ko00280
PPAR signaling pathway	69 (1.7%)	2.405392e-05	ko03320
Glyoxylate and dicarboxylate metabolism	38 (0.94%)	2.538004e-05	ko00630
Glycolysis / Gluconeogenesis	42 (1.04%)	3.846812e-05	ko00010
Citrate cycle (TCA cycle)	32 (0.79%)	4.636611e-05	ko00020
Glutathione metabolism	29 (0.72%)	8.95027e-05	ko00480
Cysteine and methionine metabolism	32 (0.79%)	9.57516e-05	ko00270
Oxidative phosphorylation	65 (1.61%)	0.0001093573	ko00190
Arginine and proline metabolism	40 (0.99%)	0.0001315773	ko00330
Starch and sucrose metabolism	38 (0.94%)	0.0005948898	ko00500
N-Glycan biosynthesis	31 (0.77%)	0.0006564914	ko00510
Aminoacyl-tRNA biosynthesis	28 (0.69%)	0.0007056859	ko00970
Glycerolipid metabolism	42 (1.04%)	0.001604464	ko00561
Pentose phosphate pathway	20 (0.49%)	0.001797754	ko00030
One carbon pool by folate	18 (0.44%)	0.003600427	ko00670
Biotin metabolism	5 (0.12%)	0.006267265	ko00780
Amino sugar and nucleotide sugar metabolism	37 (0.91%)	0.009235502	ko00520
Ubiquinone and other terpenoid-quinone biosynthesis	8 (0.2%)	0.01192872	ko00130
Nicotinate and nicotinamide metabolism	27 (0.67%)	0.02183058	ko00760
Phenylalanine, tyrosine and tryptophan biosynthesis	5 (0.12%)	0.03226023	ko00400

*, indicates the percentage of genes in each pathway from 4049 genes mapped to KEGG.

### Annotation of metabolism-relevant genes and pathways of the large yellow croaker liver

Most of the metabolism-relevant genes were significantly differentially expressed in the large yellow croaker liver after 21 d of fasting (Table 2). These genes encoding various key enzymes were found to be mainly associated with lipid metabolism (*FABP2*, *GOT2*, *NPC1L1*, *MGAT2*, *MTTP*, *APOA1*), glycometabolism (*G6PC*, *HK*, *ADPGK*, *FBP*, *pfkA*, *ALDO*, *GAPDH*, *PGAM*, *ENO*, *PEPCK*, *PK*, *LDH*, *aceE*, *DLAT*, *DLD*, *ACSS*, and *ADH1-7*), and tricarboxylic acid cycle (*MDH1*, *CS*, *ACLY*, *ACO*, *IDH3*, *fumA*, *SDHA*, *LSC1*, and *DLST*). Furthermore, several other molecules were involved in the synthesis of amino acids (glycine, serine, threonine, arginine, and proline) and fatty acids. KEGG analysis identified a large number of unigenes that were enriched in various known metabolism-relevant pathways (Table 1) such as the pathway of fat digestion and absorption, glycolysis/gluconeogenesis, citrate cycle, fatty acid metabolism, and protein processing in the endoplasmic reticulum. The remaining genes were represented by GO terms such as cytokine, apoptosis-related, and immunity genes. These biological functions and pathways have not been associated with a particular metabolism-related event.

**Table 2:** Differentially expressed genes related to metabolism-relevant pathways.

Gene name	Gene ID	Description	Fold-change	P-value
*FABP2*	Unigene24231_All	Fatty acid-binding protein 2, intestinal	-14.17	1.99E-70
*GOT2*	Unigene26274_All	Aspartate aminotransferase, mitochondrial	1.29	0
*NPC1L1*	Unigene41275_All	Niemann-Pick C1-like protein 1	2.39	6.76E-08
*MGAT2*	CL2412.Contig2_All	2-acylglycerol O-acyltransferase 2	1.05	3.08E-18
*MTTP*	CL69.Contig1_All	Microsomal triglyceride transfer protein large subunit	-2.88	0
*APOA1*	CL1084.Contig1_All	Apolipoprotein A-I	4.26	0
*G6PC*	CL2205.Contig1_All	Glucose-6-phosphatase	-5.11	1.36E-09
*HK*	Unigene40485_All	Hexokinase	11.42	3.79E-05
*ADPGK*	CL4412.Contig1_All	ADP-dependent glucokinase	-1.41	5.19E-56
*FBP*	Unigene16513_All	Fructose-1,6-bisphosphatase I	-1.18	2.20E-05
*pfkA*	CL830.Contig1_All	6-Phosphofructokinase 1	-1.12	3.16E-06
*ALDO*	Unigene1497_All	Fructose-bisphosphate aldolase, class I	4.35	0
*GAPDH*	CL2528.Contig1_All	Glyceraldehyde 3-phosphate dehydrogenase	2.82	0
*PGAM*	CL1161.Contig2_All	2,3-Bisphosphoglycerate-dependent phosphoglycerate mutase	-1.73	1.21E-37
*ENO*	CL4254.Contig1_All	Enolase	3.05	3.17E-194
*PEPCK*	Unigene36521_All	Phosphoenolpyruvate carboxykinase	-11.58	3.24E-09
*PK*	Unigene13089_All	Pyruvate kinase	1.24	1.92E-27
*LDH*	Unigene10413_All	L-Lactate dehydrogenase	-2.18	1.27E-08
*aceE*	Unigene16826_All	Pyruvate dehydrogenase E1 component	-2.64	0
*DLAT*	Unigene10467_All	Dihydrolipoamide acetyltransferase	-1.64	8.89E-12
*DLD*	Unigene16721_All	Dihydrolipoamide dehydrogenase	1.42	2.03E-186
*ACSS*	Unigene10099_All	Acetyl-CoA synthetase	-2.94	6.90E-59
*ADH1_7*	Unigene7936_All	Alcohol dehydrogenase 1/7	2.61	0
*MDH1*	Unigene42274_All	Malate dehydrogenase	2.56	1.15E-05
*CS*	Unigene17567_All	Citrate synthase	-3.72	0
*ACLY*	CL1940.Contig1_All	ATP citrate (pro-S)-lyase	-4.46	0
*ACO*	CL2020.Contig1_All	Aconitate hydratase	-1.35	8.66E-156
*IDH3*	Unigene14609_All	Isocitrate dehydrogenase (NAD+)	-1.97	1.28E-113
*fumA*	Unigene29909_All	Fumarate hydratase, class I	1.18	3.61E-27
*SDHA*	Unigene16872_All	Succinate dehydrogenase (ubiquinone) flavoprotein subunit	-2.82	1.19E-17
*LSC1*	CL362.Contig2_All	Succinyl-CoA synthetase alpha subunit	-1.85	3.43E-24
*DLST*	Unigene40361_All	2-Oxoglutarate dehydrogenase E2 component	1.45	5.37E-08

Identification of all differentially expressed genes was based on *P* < 0.05. A P value < 0.05 indicated that the gene was significantly altered in fasting fish relative to that observed in normal feeding fish. The absolute value of “Fold-change” is the magnitude of up- or downregulation for each gene after fasting. “+” indicates upregulation, and “-” indicates downregulation.

### Differentially expressed unigenes in the glycolysis/gluconeogenesis, fat digestion and absorption, and TCA cycle pathways

**Fig 2:**
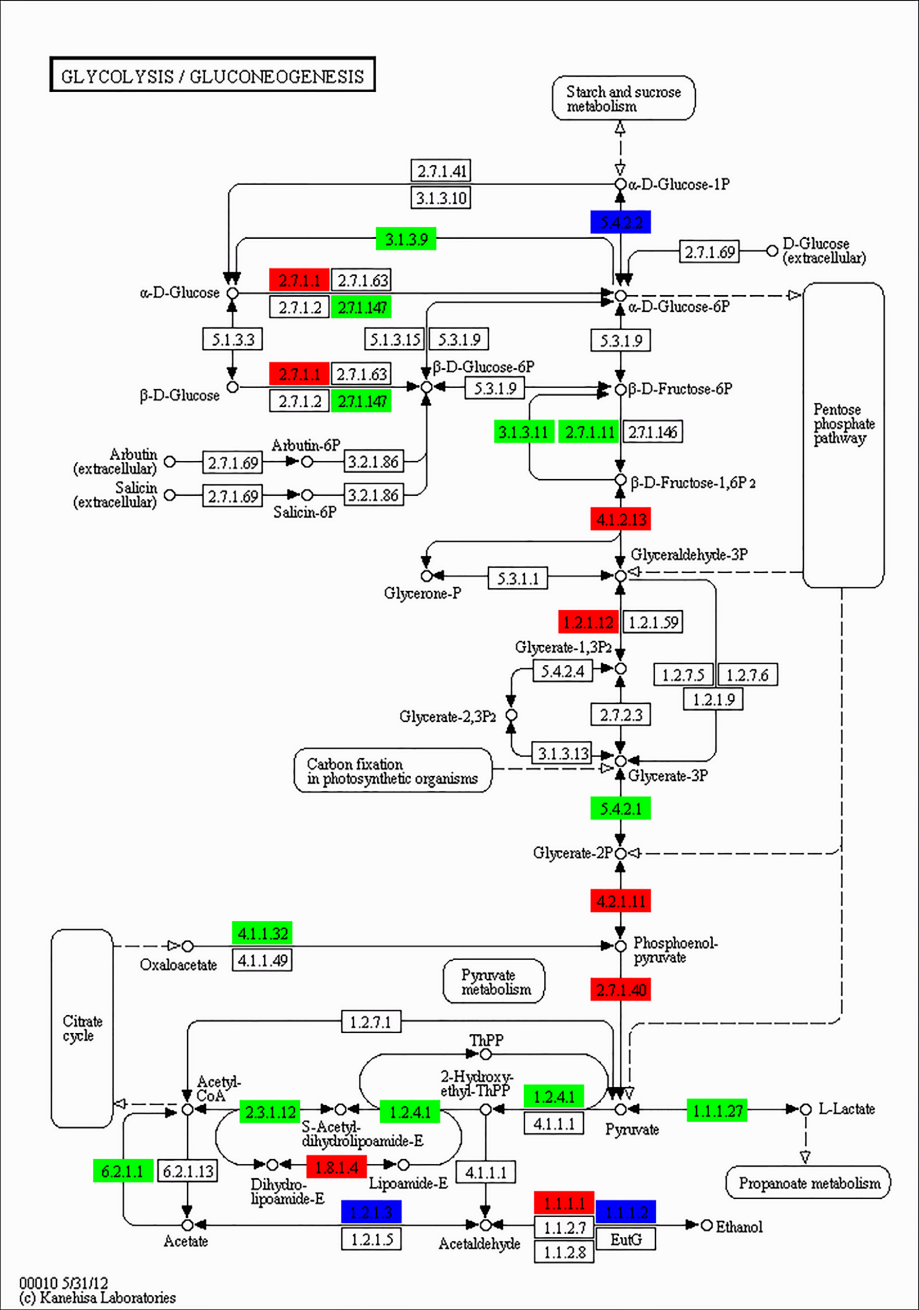
Significantly differentially expressed genes identified by KEGG in the glycolysis/gluconeogenesis pathway. Red indicates significantly upregulated genes, green indicates significantly downregulated genes, and blue indicates genes that were both up- and downregulated.

**Fig 3:**
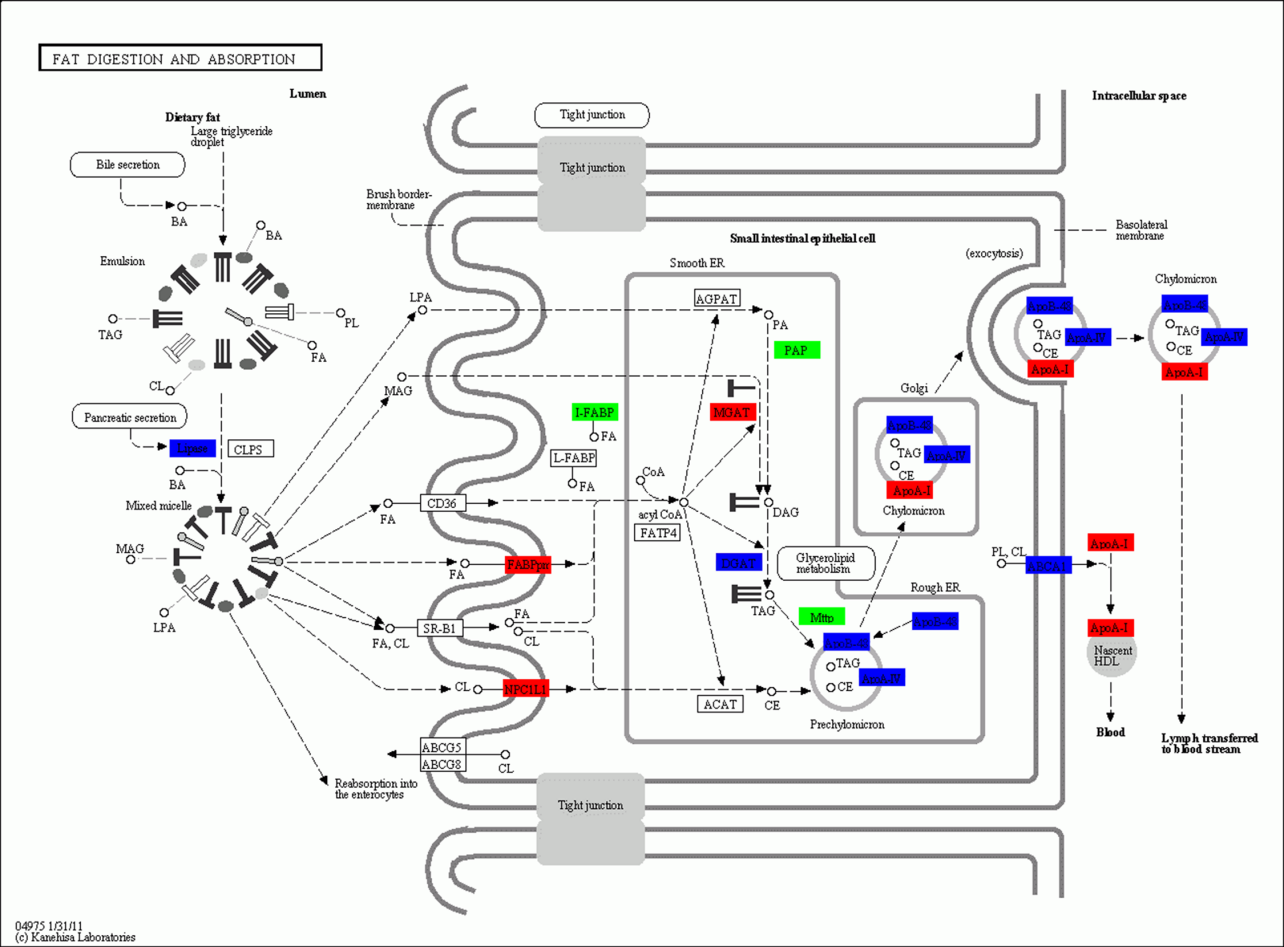
Significantly differentially expressed genes identified by KEGG in the pathway of fat digestion and absorption. Red indicates significantly upregulated genes, green indicates significantly downregulated genes, and blue indicates genes that were both up- and downregulated.

**Fig 4:**
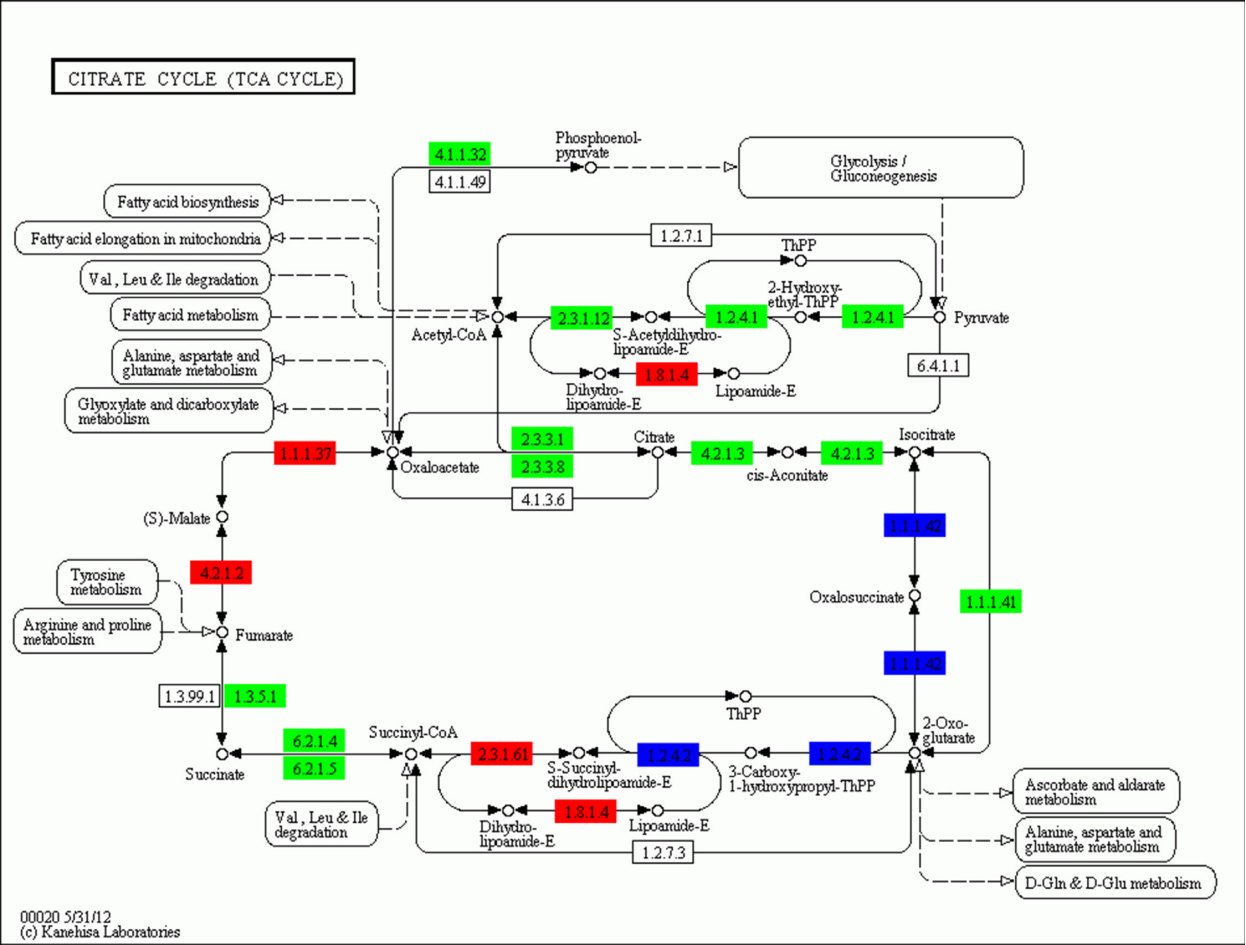
Significantly differentially expressed genes identified by KEGG in the TCA cycle pathway. Red indicates significantly upregulated genes, green indicates significantly downregulated genes, blue indicates genes that were both up- and downregulated.

To further explore the physiological response profiles induced by fasting at a single-pathway level, KEGG analysis was performed to identify genes involved in the metabolic pathway. In the present study, a large number of differentially expressed genes with functions in glycolysis, gluconeogenesis, and fat digestion and absorption were upregulated or downregulated in the liver of the large yellow croaker following 21 d of fasting. In the pathway of glycolysis/gluconeogenesis (**Fig 2**), seven genes were identified as highly upregulated after 21 d of fasting, *HK*, *ALDO*, *GAPDH*, *ENO*, *PK*, *DLD*, and *ADH*1-7. On the other hand, 11 genes were highly downregulated in response to fasting after 21 days, including *G6PC*, *ADPGK*, *FBP*, *pfkA*, *PGAM*, *PEPCK*, *ACSS*, *DLAT*, *aceE*, and *LDH*. 4 genes (*FABP*, *NPC1L1*, *MAGAT*, and *ApoB-I*) were remarkably upregulated and 3 genes (*I-FABP*, *PAP*, and *Mttp*) markedly downregulated in the fat digestion and absorption pathway (**Fig 3**). 4 significantly upregulated genes (*fumA*, *MDH1*, *DLST* and *DLD*), and 10 significantly downregulated genes (*PEPCK*, *DLAT*, *aceE*, *CS*, *ACLY*, *ACO*, *SDHA*, *LSC1*, *sucD*, and *IDH*3) were found in the TCA cycle (**Fig 4**). In addition, unlike the genes involved in fat digestion and absorption, genes encoding proteins that participate in fatty acid metabolism were upregulated, except for *ACOX1*.

In skeletal muscle transcriptome after 21 days fasting, differential expressions of genes in TCA cycle were most significant with KEGG analysis. In TCA cycle, the expression patterns of some genes were similar to that of liver. For example, *HK*, *ENO* and *PK* were upregulated, and *G6PC*, *ADPGK*, *FBP*, *pfkA*, *PGAM* and *LDH* were downregulated in the pathway of glycolysis/gluconeogenesis (S1 Fig). In the TCA cycle, genes *DLST* and *DLD* were significantly upregulated, and *ACO*, *LSC1*, *sucD* were downregulated (S2 Fig). In the pathway of the fat digestion and absorption (S3 Fig), both *PAP* and *I-FABP* were upregulated. However, expression pattern of some genes was different between liver and skeletal muscle. In the pathway of the fat digestion and absorption, *CD36* and *SR-B1* were upregulated in skeletal muscle, and kept constant in the liver; *MGAT* and *Mttp* were upregulaten in skeletal muscle, and downregulated in the liver; *Lipase* mRNA increased in skeletal muscle, and fluctuated in the liver.

### Protein and amino acid metabolism

**Fig 5:**
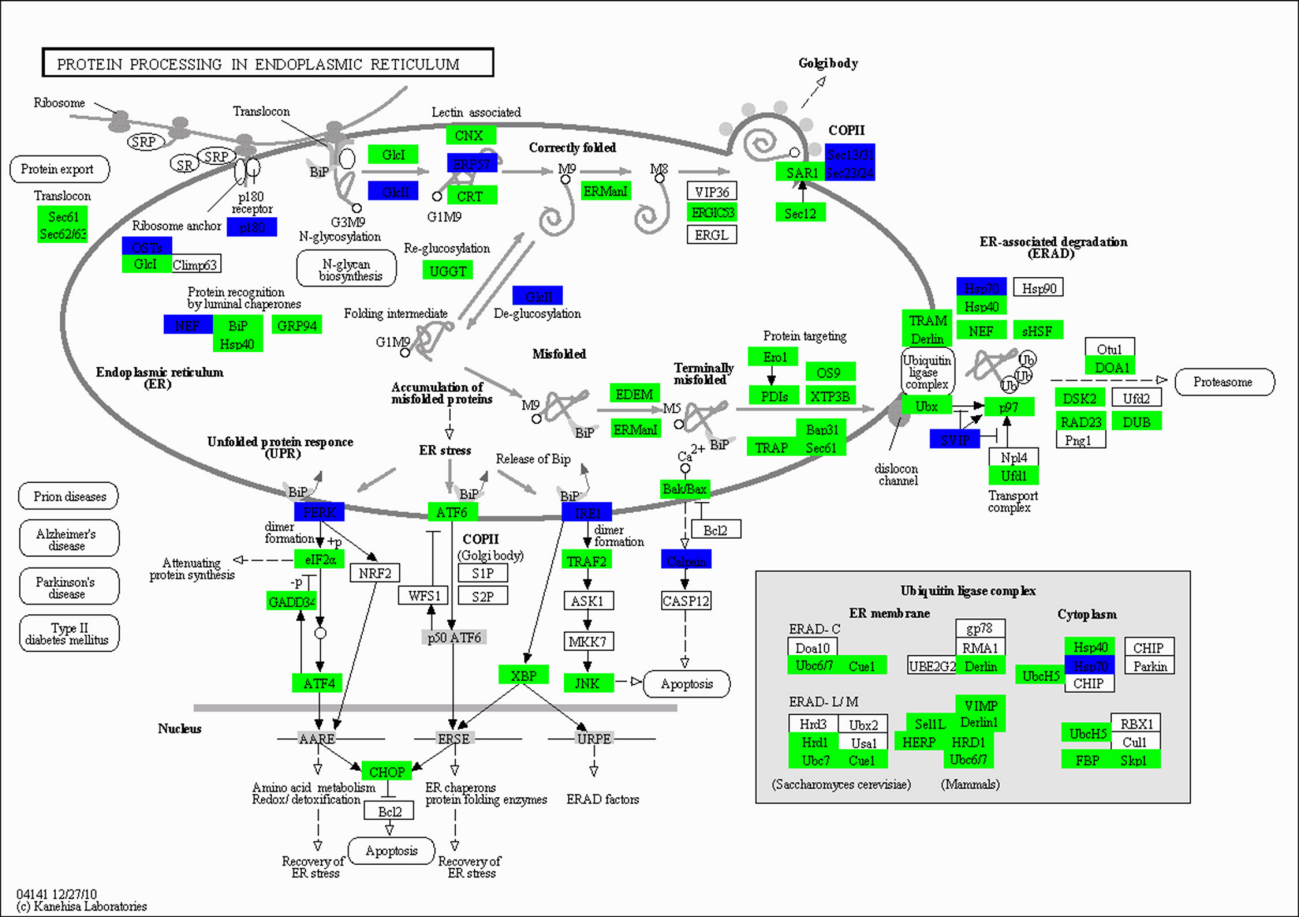
Significantly differentially expressed genes identified by KEGG in the protein processing in the endoplasmic reticulum pathway. Green indicates significantly downregulated genes, and blue indicates genes that were both up- and downregulated.

RNA-seq analysis revealed that a large number of genes related to protein synthesis and protein degradation underwent significant changes in expression levels following 21 d of fasting. In the protein processing in the endoplasmic reticulum pathway, all annotated unigenes (e.g., *Sec61*, *SAR1*, *eIF2a*, and *ATF6*) were downregulated (**Fig 5**). A similar change in expression was observed in genes related to the protein export pathway such as genes encoding signal recognition particle subunits (*SRP9*, *SRP72*, *SRP19*, *SRP14*, *SRP68*, *SRP54*, *SRPR*, and *SRPRB*), genes encoding protein transport proteins (*SEC61α*, *SEC61β*, *SEC61γ*, *SEC62*, *SEC63*, and *SEC11*), and genes coding for signal peptidase complex subunits (*SPCS1* and *SPCS2*). Ultimately, most of genes encoding proteins related to amino acid catabolism (e.g., valine, leucine, isoleucine, arginine, and proline) were upregulated in the liver of fasting large yellow croakers.

### qPCR analysis of key genes in the glycolysis/gluconeogenesis, TCA cycle, and fat digestion and absorption pathways

**Fig 6:**
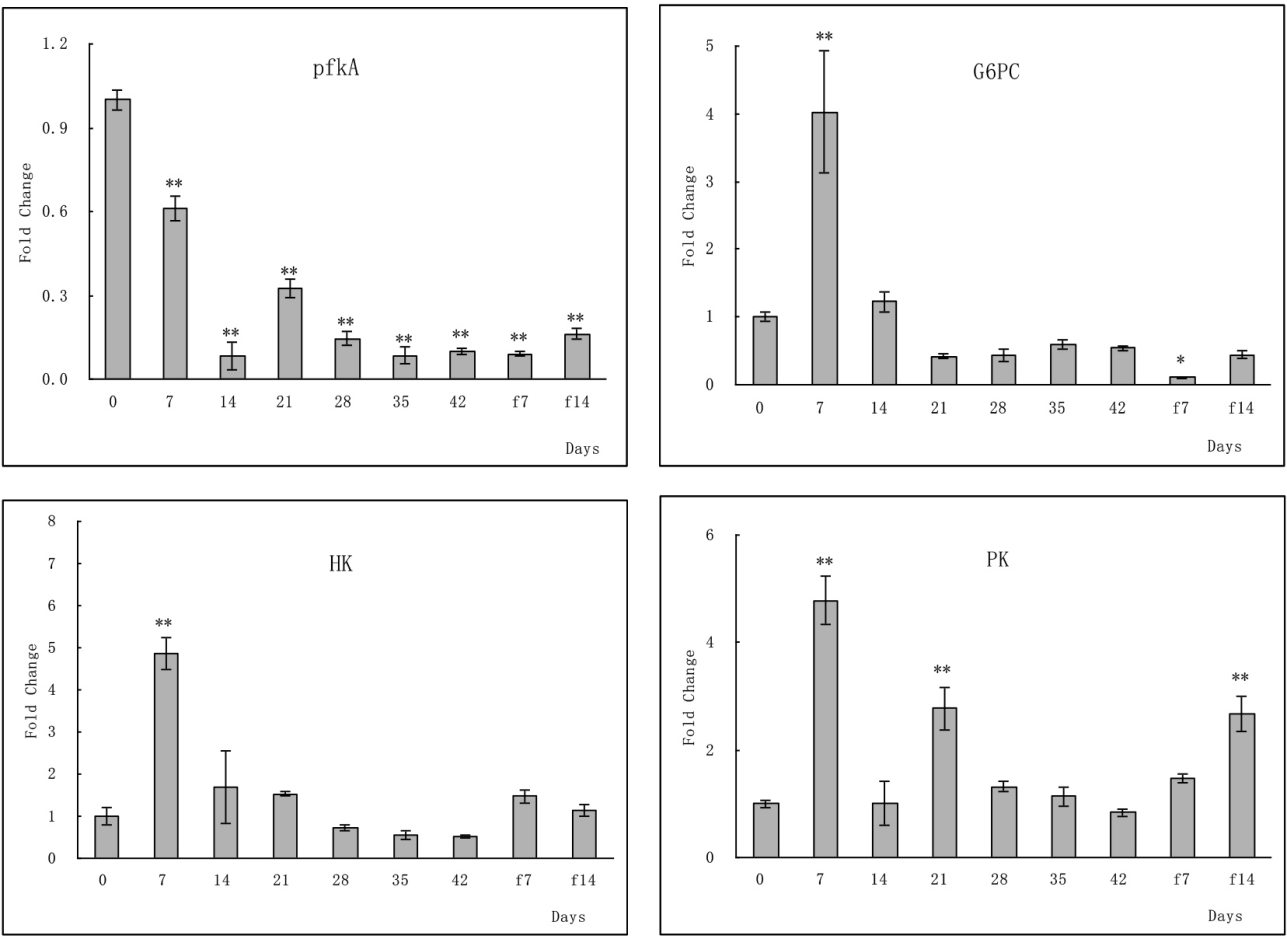
qPCR analysis of genes related to the glycolysis/gluconeogenesis pathway in the large yellow croaker during fasting and re-feeding. To determine changes in the levels of expression of the following genes, 6-phosphofructokinase 1(*pfkA*), glucose-6-phosphatase (*G6PC*), hexokinase (*HK*), and pyruvate kinase (*PK*), the livers of 6 fish were collected at 0, 7, 14, 21, 28, 35, 42 days of fasting and 7 and 14 days of re-feeding, respectively. Total RNA was extracted from these livers and used in qPCR analysis. The mRNA level of each gene was normalized to that of β-actin. For each time point, values represent fold change in expression of each gene compared to that observed at 0 d fasting, which was set at 1.0. The results are expressed as means ± SD (n = 3). Independent-sample *t*-test using the SPSS software (Version 11.5) was conducted to determine the statistical significance of the changes in expression levels in fasting or re-feeding fish relative to that observed in 0-d fasting fish. Significant differences were considered at *0.01 < *p* ≦ 0.05 and ** *p* < 0.01.

**Fig 7:**
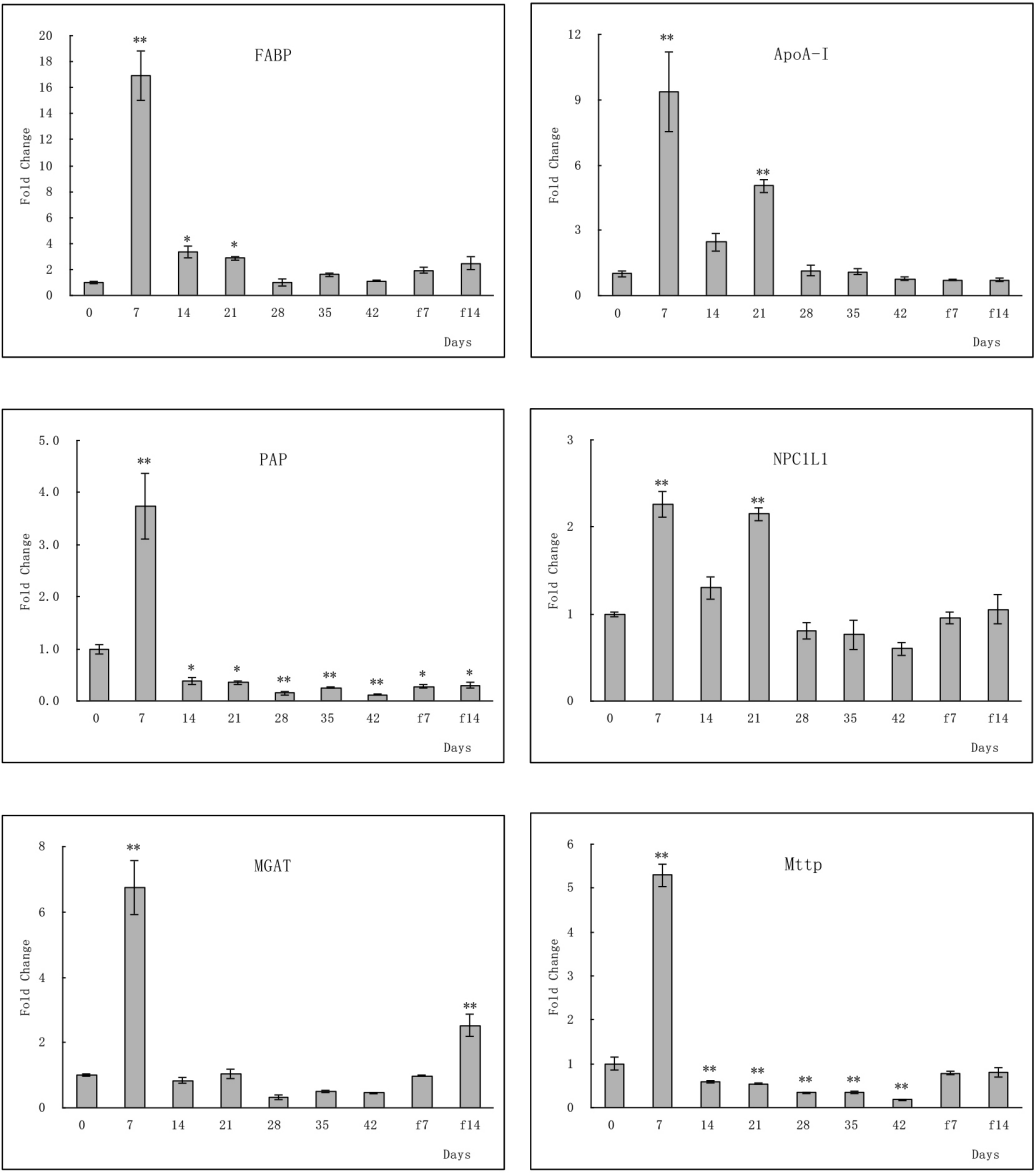
qPCR analysis of the genes related to the fat digestion and absorption pathway in the large yellow croaker during fasting and re-feeding stages, namely, fatty acid-binding protein (*FABP*), apolipoprotein A-I (*ApoA-I*), phosphatidate phosphatase (*PAP*), Niemann-Pick C1-like protein 1 (*NPC1L1*), 2-acylglycerol O-acyltransferase (*MGAT*), microsomal triglyceride transfer protein large subunit (*Mttp*). The experimental procedure was similar to that presented in Fig 6.

**Fig 8:**
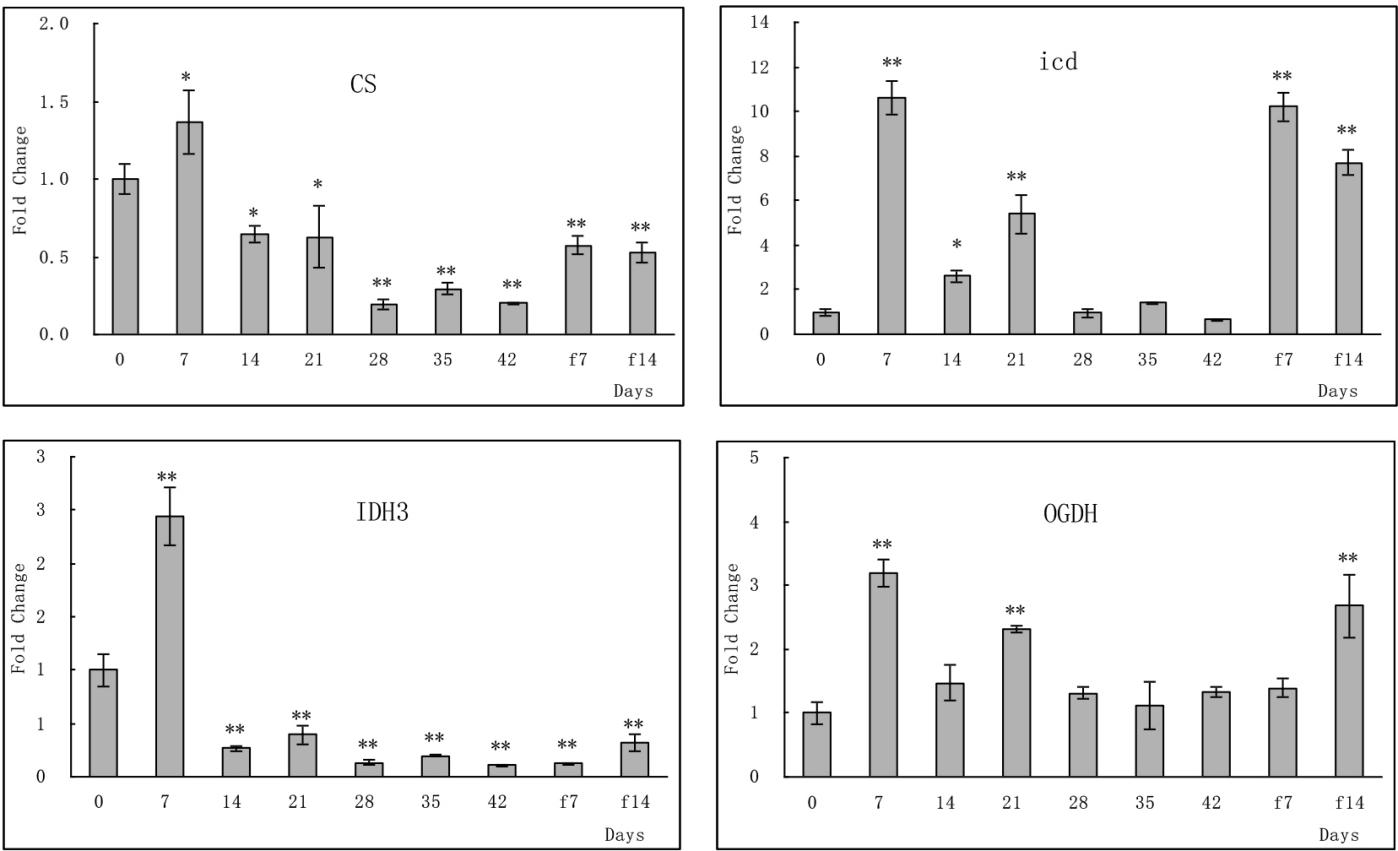
qPCR analysis of the genes related to the TCA cycle pathway in the large yellow croaker during fasting and re-feeding stages, namely, citrate synthase (*CS*), isocitrate dehydrogenase (*icd*), isocitrate dehydrogenase (*IDH3*), 2-oxoglutarate dehydrogenase E1 component (*OGDH*). The experimental procedure was similar to that presented in Fig 6.

To further clarify the strength of the correlations of the up- or downregulated genes to the pathway of fat digestion and absorption, glycolysis/gluconeogenesis, and TCA cycle in the large yellow croaker during the stages of fasting and re-feeding, differential expression of 14 genes was analyzed by qPCR. This experiment was also conducted to confirm the hypothesis that the glycol and fat digestion and absorption pathways elicit an adaptive response to fasting and re-feeding. The assay was performed using the large yellow croaker liver samples collected after 42 d of fasting followed by 14 d of re-feeding. Most of the results were in line with those of the RNA-seq analysis (**Figs 6–8**). The key genes *pfkA* and *PK* in the glycolysis/gluconeogenesis pathway that were expressed in liver tissues from the experimental groups were significantly regulated relative to that of the normal feeding group. The genes *HK* and *G6PC* showed no significant change in the levels of gene expression after 21 days of fasting (**Fig 6**). In the TCA cycle pathway, genes coding for key enzymes were significantly regulated after fasting and re-feeding (**Fig 8**). Significant changes in the level of expression of genes related to fat digestion and absorption were observed in the large yellow croaker liver tissues compared to that observed in the control groups, except for gene *MGAT* (**Fig 7**).

## Discussion

Molecular studies on metabolism during fasting in the large yellow croaker are limited, and there is currently no report that describes the transcriptome profile of the liver during this particular condition. To increase our knowledge on the effect of fasting on gene expression in the large yellow croaker, the transcriptome profile of the fish after fasting was analyzed. In this study, *de novo* assembly tools [15] were used to assemble the short reads generated by RNA-seq, which in turn generated pertinent sequence information. Quantitative gene expression profiling was also conducted, and the unigenes were annotated and mapped to the generated transcriptome database. Several differentially expressed genes were involved in glycol, lipid, and protein metabolism such as those encoding key enzymes involved in fatty acid metabolism, amino degradation, and other biological functions.

Glucose homeostasis is precisely regulated to meet the energy demands during the fasting-re-feeding cycle in fish. Glycolysis and gluconeogenesis are opposing metabolic pathways involved in the breakdown and synthesis of carbohydrates in the liver and are essential for fish survival particularly during prolonged periods of starvation [27]. Most enzymes are shared by the glycolysis and gluconeogenesis pathways, except for three key enzymes, GK, pfkA, and PK, which catalyze unidirectional reactions in glycolysis, and another three enzymes, PEPCK, FBP, and G6PC, which induce the reverse reactions and play a key role in gluconeogenesis. In the present study, differentially expressed unigenes related to glucose metabolism were annotated in the KEGG pathway, which revealed significant changes in the expression levels of most genes coding for enzymes involved in the glycolysis/gluconeogenesis pathway. For example, the expression of key genes *PEPCK*, *FBP*, and *G6PC* were downregulated after 21 d of fasting, and it was also occurred in skeletal muscle transcriptome responding to fasting. This finding was also observed by Metón *et al*. in the gilthead seabream, wherein the value of PK and G6P-DH enzyme activities were significantly decreased after 18 days of starvation [28]. On the other hand, the *PK* gene was significantly upregulated in the glycolysis/gluconeogenesis pathway in the present study, and it was same to the result of skeletal muscle transcriptome analysis. Similar results have been reported in the muscle of the rainbow trout after 30 days of starvation [29].

Fish utilize lipids as a major source of energy [30]. In the present study, transcriptome analysis revealed that fasting led to a dramatic increase in the expression of genes encoding for proteins involved in fatty acid metabolism such as *acd*, *echA*, and *HADH*. These results suggested a reallocation of lipid reserves and cholesterol metabolism, as well as demonstrated that there was an attempt to increase lipid metabolism during fasting. Studies have shown that several genes were upregulated in the liver of the Atlantic salmon following starvation [31]. Fasting fish showed an increased mRNA expression of *FABPpm*, *NPC1L1*, *MGAT*, *ApoB-I* mRNA, whereas that of *PAP*, *Mttp*, and *I-FABP* was reduced, which were confirmed by qPCR analysis. Apolipoproteins, which are plasma lipoprotein complexes, were upregulated in the liver of various fish species; these bind to lipids, which are then transported to different tissues through the blood [32]. *FABP* binds and transports long-chain fatty acids from the aqueous cytoplasm to the site of its oxidation in the mitochondria [33]. Furthermore, other up- or downregulated genes involved in the pathway of fat digestion and absorption were suggestive that hepatic fatty acid metabolism was active after fasting. Hepatic fat is the first storage tissue mobilized during fasting and perhaps the most important fat depot for energy in the large yellow croaker. Expression of some genes relate to fat-metabolism was up-regulated during fasting, and still up-regulated after 14 days re-feeding in the liver, which might implied that physiological status of the fasted fish had not recoverd to normal level after re-feeding of short time. In addition, some genes encoding protein CD36 and SR-B1 were upregulated significantly in the pathway of fat digestion and absorption in skeletal muscle, and were not changed in the liver, which implied the different fat metabolism between liver and skeletal muscle, and the delay of fat digestion in skeletal muscle. *Lipase* was upregulated significantly in the skeletal muscle, which confirmed that fasting could accelerate the consumption of fat.

Most proteins are synthesized and degraded in the liver [31]. During fasting, the liver receives a smaller amount of free amino acids that are derived from digestion [34], thus resulting in a decline in the synthesis of proteins in the liver [35]. Although rates of both synthesis and degradation are decreased during fasting, these two reactions are tightly regulated. In the present study, transcriptome analysis after 21 d of fasting revealed that the levels of protein synthesis in the endoplasmic reticulum and protein transport significantly decreased, whereas the rate of amino acid catabolism increased, similar to that observed in the rainbow trout [31]. The molecules generated by the breakdown of amino acids may be contribute to gluconeogenesis and subsequently enter the TCA cycle. The TCA cycle is an important aerobic pathway for the final of carbohydrate and fatty acid oxidation [36]. The present study showed that several genes encoding enzymes involved in the TCA cycle were downregulated, except for the *fum*A-B, *MDH1*, *DLST*, and *DLD* genes, which were upregulated. MDH1 is a key enzyme in the catalysis of oxaloacetate, and during fasting the increase in the production of oxaloacetate may be a response to the decrease in the levels of alanine, aspartate, and glutamate. Furthermore, the decrease in the level of gene expression of *DLAT* may lead to a reduction in the synthesis of acetyl-CoA, which in turn leads to the upregulation of genes coding for enzymes in the valine, leucine, and isoleucine degradation and fatty acid metabolism pathways.

## Conclusions

The present study conducted a transcriptional profiling analysis of fasting and normal feeding large yellow croakers to identify metabolism-related genes and pathways. RNA-seq data revealed major changes in the expression of metabolic genes in the fasting large yellow croaker, thus providing insights into the molecular mechanism underlying this biochemical reaction in fish. Additional studies using the transcriptome data generated in the present study will likely provide further significant insights into the detailed mechanisms of fasting in the large yellow croaker, which may in turn benefit the aquaculture industry, particularly in terms of propagating this economically important marine species.

## Materials and Methods

### Ethics Statement

The protocols used in the present study were in accordance with the “Regulations for the Administration of Affairs Concerning Experimental Animals” that was established by the Zhejiang Provincial Department of Science and Technology on the Use and Care of Animals. Animal experiments were approved by the Animal Care and Use Committee of Ningbo University. And to minimize suffering, all surgeries of this experiment were performed Tricaine-S anesthesia. In this study, all experiment fish caught from net-cages were anesthetized by Tricaine-S (TMS, MS-222) firstly (*50 mg/L*). We collected the liver and skeletal muscle samples after the anesthetized fish has lost consciousness. The fish which has been taken a surgery would be sprayed with anesthetic (200 mg/L), and let it death with euthanasia.

### Fish and fasting experiments

Large yellow croakers (mean weight: 68±0.8 g) were purchased from Xiangshan bay aquatic fingerlings company, Zhengjiang, China, on September 27. The fish were randomly cultured in 10 net-cages and the temperature of the sea water was 22°C. During first 7 days, fish was fed twice a day, at 5 o’clock in the morning and at half past 6 in the evening, with surimi made by Xiangshan bay aquatic fingerlings company until the fish stopped eating. After 7 days of acclimation, the fish was used to the fasting experiments. In this experiment, the fish had been fasted (without food entirely) for 42 days, and then re-feeding 14 days. Livers and skeletal muscle were collected from 6 fish at each time point at 0 day and every 7 days, and then stored in the storage medium, RNAstore (CWBIO, China), until RNA extraction and transcriptome analysis. All experiments were conducted at the Xiangshan Bay and Ningbo University, Zhejiang, China.

### RNA isolation

Total RNA was extracted from 30 mg to 50 mg of tissue using the tissue RNA kit (Omega, China), following the manufacturer’s instructions. The quality and quantity of RNA samples were determined by measuring its absorbance at 260/280 nm (A 260/280) using an Ultrospec 1100 pro spectrophotometer (Healthcare Bio-Sciences AB, Sweden). The average RIN of samples was 7.5, as indicated by the Agilent 2100 Bioanalyzer (Agilent Technologies, USA).

### Library preparation and sequencing

For gene expression profiling, mRNA was extracted from the livers of fasting 21 days and normal-feeding large yellow croakers (six individuals in each group) using oligo (dT) magnetic beads, mixed with a fragmentation buffer, and subjected to fragmentation. Using the fragmented mRNA as template, cDNA was then synthesized. The short fragments were purified and resolved with EB buffer for end-repair and single nucleotide A (adenine) addition, then connected to adapters. Suitable fragments were selected as templates for PCR amplification. For QC, the Agilent 2100 Bioanalyzer and ABI StepOnePlus Real-Time PCR System were used in the quantification and qualification of the sample library. Finally, the libraries were sequenced on an Illumina HiSeq^TM^ 2000 system. The raw data files were passed to NCBI's Sequence Read Archive (SRA), and the accession number is SRX986569 (LB2A), SRX986572 (LF1A).

### Transcript assembly and annotation

To obtain unigenes, transcriptome *de novo* assembly was conducted using the short-read assembly program, Trinity (release-20121005). Then, BLASTx alignment (e-value < 0.00001) between unigenes and protein databases such as NR, Swiss-Prot, KEGG, and COG was performed, and the best alignment results were used to establish the sequence direction of unigenes. With NR annotation, the Blast2GO program was used to perform GO annotation of the unigenes, and the KEGG Automatic Annotation Server (KAAS) system was used for pathway reconstruction. All of data have been submitted to the NCBI GEO database, and the series record is GSE67756 (http://www.ncbi.nlm.nih.gov/geo/query/acc.cgi?acc=GSE67756).

### Quantitative real-time PCR (qPCR) analysis

qPCR analysis was performed using the Mastercycler epgradient realplex (Eppendorf, Germany with SYBR Green (Roche, USA) as the fluorescent dye and following the manufacturer’s protocol. The primers were designed based on each identified gene sequence of the liver transcriptome library by using Primer Premier 5.0 (S2 Table). Prior to qPCR analysis, every primer was PCR validated, which indicated that no primers showed dimmers in its melting curves and a single band was observed on the agarose gels (S4 Fig). Total RNA was extracted from the liver tissues of six large yellow croakers that were sampled at 0, 7, 14, 21, 28, 35, 42 d after fasting, and 7 and 14 d of re-feeding. First-strand cDNA was synthesized from 1 μg of total RNA by using PrimeScript^TM^ RT reagent Kit (Takara, Japan), which was then used as template for qPCR analysis using gene-specific primers. qPCR analysis was performed using a total volume of 20 μL, and the cycling conditions were as follows: 95°C for 10 min, followed by 40 cycles of 95°C for 15 s, 59°C for 40 s, and 72°C for 20 s. Each qPCR reaction was performed in triplicate, and the data of each sample were expressed relative to the expression levels of β-actin by using the 2^-ΔΔCT^ method [37]. Statistical significance was determined by using independent-sample *t*-test as provided in the SPSS software (Version 21), and significant differences were determined at *P* < 0.05.

## Supporting Information

S1 Fig Significantly differentially expressed genes identified by KEGG in the glycolysis/gluconeogenesis pathway of the skeletal muscle transcriptome analysis. Red indicates significantly upregulated genes, green indicates significantly downregulated genes, pale red indicates genes that were upregulated but not significantly, and light grey indicates genes that were downregulated but not significantly.
(TIF)

S2 Fig Significantly differentially expressed genes identified by KEGG in the TCA pathway of the skeletal muscle transcriptome analysis. Red indicates significantly upregulated genes, green indicates significantly downregulated genes, pale red indicates genes that were upregulated but not significantly, and light grey indicates genes that were downregulated but not significantly.
(TIF)

S3 Fig Significantly differentially expressed genes identified by KEGG in the fat digestion and absorption pathway of the skeletal muscle transcriptome analysis. Red indicates significantly upregulated genes, green indicates significantly downregulated genes, pale red indicates genes that were upregulated but not significantly, and light grey indicates genes that were downregulated but not significantly.
(TIF)

S4 Fig Agarose gel electrophoresis of primers validation. Namely, β-actin, 6-phosphofructokinase 1(*pfkA*), glucose-6-phosphatase (*G6PC*), hexokinase (*HK*), and pyruvate kinase (*PK*) fatty acid-binding protein (*FABP*), apolipoprotein A-I (*ApoA-I*), phosphatidate phosphatase (*PAP*), Niemann-Pick C1-like protein 1 (*NPC1L1*), 2-acylglycerol O-acyltransferase (*MGAT*), microsomal triglyceride transfer protein large subunit (*Mttp*) citrate synthase (*CS*), isocitrate dehydrogenase (*icd*), isocitrate dehydrogenase (*IDH3*) 2-oxoglutarate dehydrogenase E1 component (*OGDH*).
(TIF)

S1 Table Details on 7624 differentially expressed genes in expression profile of large yellow croaker. The data show the 7624 unigenes that were differentially expressed in the 21 d fasting and normal large yellow croaker.
(XLS)

S2 Table Primers for quantitative real-time PCR. (DOC)
