# Supplementary figures and images for: Liver Transcriptome Analysis of the Large Yellow Croaker (Larimichthys crocea) during Fasting by Using RNA-Seq
Source: PLoS One. 2016 Mar 11;11(3):e0150240. doi: 10.1371/journal.pone.0150240 (PMC4788198; doi:10.1371/journal.pone.0150240)

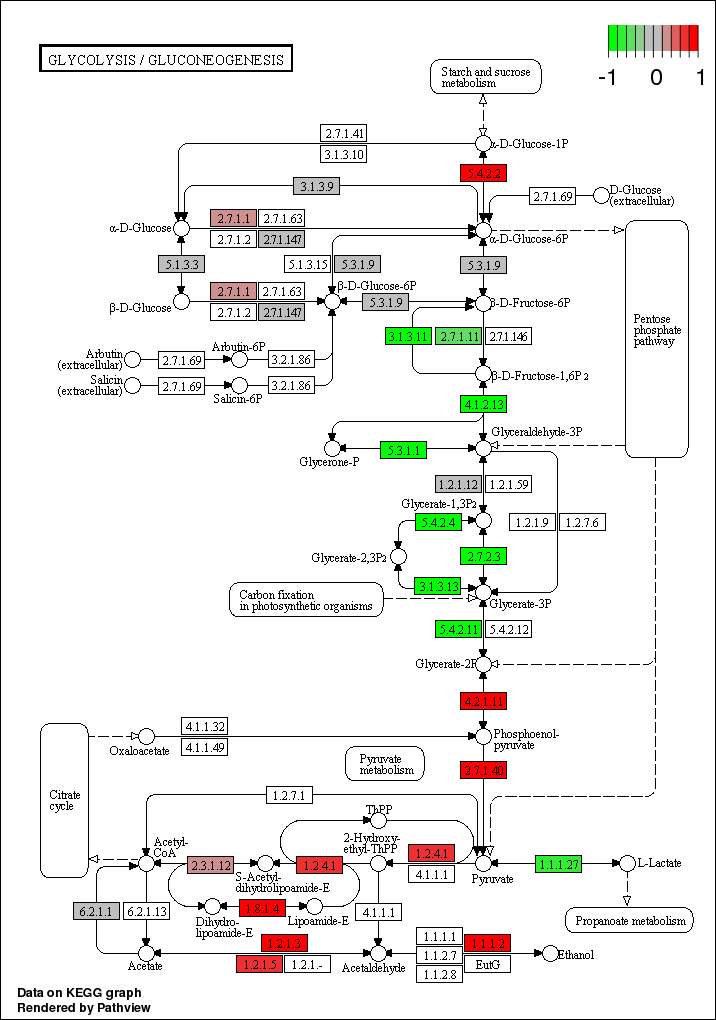

Supplement: S1 Fig — Red indicates significantly upregulated genes, green indicates significantly downregulated genes, pale red indicates genes that were upregulated but not significantly, and light grey indicates genes that were downregulated but not significantly. (TIF) [file pone.0150240.s001.tif]

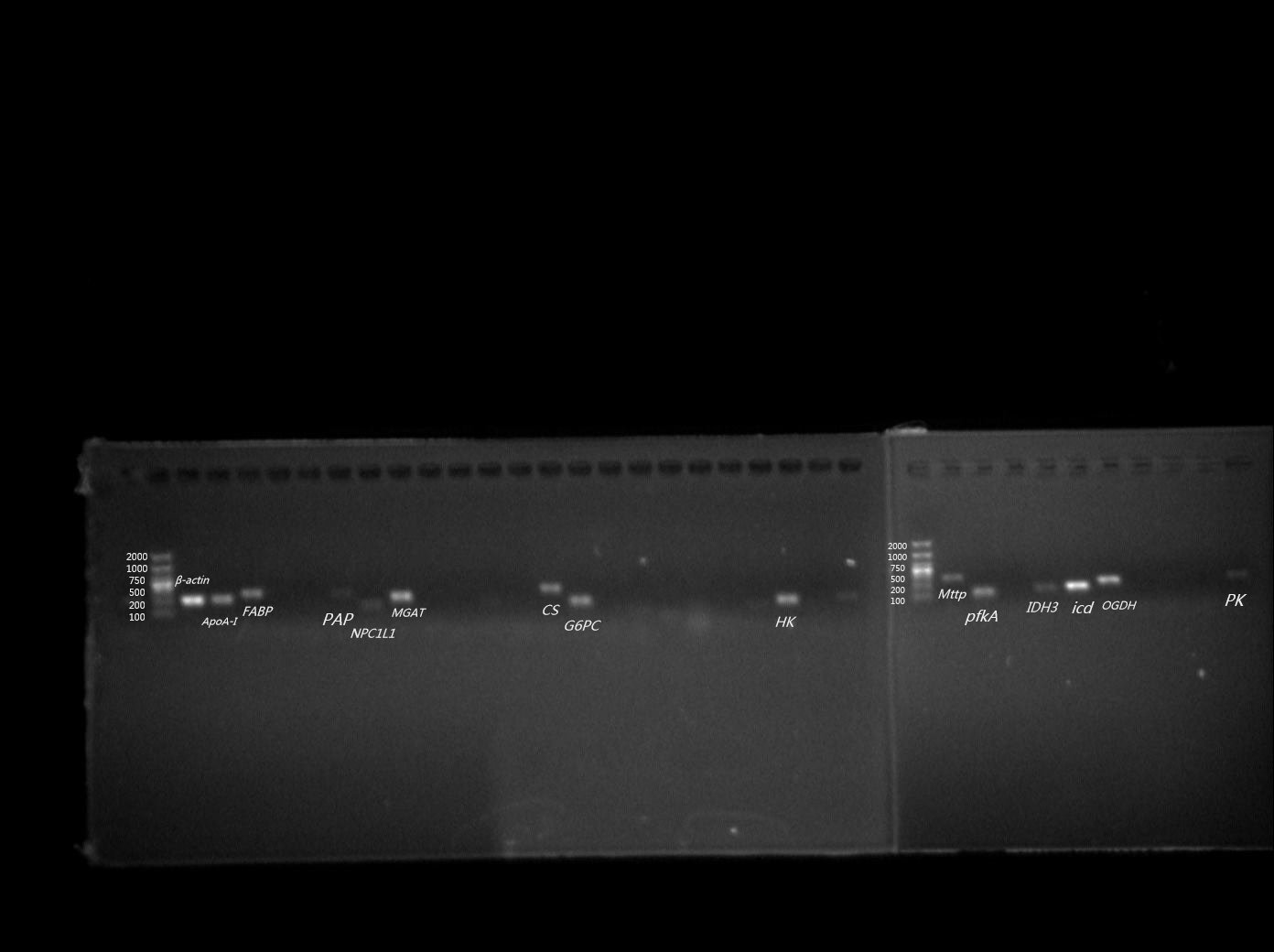

Supplement: S4 Fig — Namely, β-actin, 6-phosphofructokinase 1(pfkA), glucose-6-phosphatase (G6PC), hexokinase (HK), and pyruvate kinase (PK) fatty acid-binding protein (FABP), apolipoprotein A-I (ApoA-I), phosphatidate phosphatase (PAP), Niemann-Pick C1-like protein 1 (NPC1L1), 2-acylglycerol O-acyltransferase (MGAT), microsomal triglyceride transfer protein large subunit (Mttp) citrate synthase (CS), isocitrate dehydrogenase (icd), isocitrate dehydrogenase (IDH3) 2-oxoglutarate dehydrogenase E1 component (OGDH). (TIF) [file pone.0150240.s004.tif]
