# Supplementary material for: Liver Transcriptome Analysis of the Large Yellow Croaker (Larimichthys crocea) during Fasting by Using RNA-Seq
Source: PLoS One. 2016 Mar 11;11(3):e0150240. doi: 10.1371/journal.pone.0150240 (PMC4788198; doi:10.1371/journal.pone.0150240)
Supplement: S2 Table — (DOC) [file pone.0150240.s006.doc]

S2 Table. Primers for quantitative real-time PCR.

| Gene | Forward primer or reverse primer | Primer（5-3） |
| --- | --- | --- |
| FABP | F | CCACATCCTACCAGGCTCTA |
|  | R | TGCCCTGGTCATCCCTGT |
| NPC1L1 | F | AGGTGGTGGTGAGGAATG |
|  | R | CCAGAGCCAGACAGTAAAG |
| PAP | F | AAGGTCTTGAAGTGGAGGGT |
|  | R | GCAGGTTTGGGTTTCACA |
| MGAT | F | GAACAGACAGGGAGCGAAAC |
|  | R | TGCCCGTCTACTCATTCG |
| Mttp | F | GTCAATAGCCAACCCTCC |
|  | R | TCTTGAGTCGCTGATTGC |
| ApoA-I | F | TTTCTGAGCGTCTGGAGG |
|  | R | TGGGAGGATTTAGTTCTTGG |
| HK | F | GGGAAAGGATGTAGTCAGC |
|  | R | TCATCGTAGCCACAGGTC |
| pfkA | F | AGTTCGGATTCCAGTTTACG |
|  | R | TGTGGAGGCTGTTCTTGC |
| PK | F | GTGAAGGGAATGGAGACG |
|  | R | TCCCTCCACTTTCATCTATC |
| G6PC | F | TCCACGAGGCATCTTATT |
|  | R | TGGTCATTATGGCGAGGA |
| CS | F | GTGGGAGGAAGTGGAAAC |
|  | R | TCTGAGCCTACGATGGGT |
| icd | F | GGATAAGGCAGTGAGGGT |
|  | R | TGATGGCTCAGTGACCTT |
| OGDH | F | TCTGGAGGGCTGTGAAGT |
|  | R | TCTGGAGGGCTGTGAAGT |
| IDH3 | F | AAGACCCGTCACCACTCA |
|  | R | CAGAAAGCATTGGCACCC |
